# Subthalamic stimulation evokes hyperdirect high beta interruption and cortical high gamma entrainment in Parkinson’s disease

**DOI:** 10.1038/s41531-025-00965-6

**Published:** 2025-04-26

**Authors:** Ádám József Berki, Hao Ding, Marcell Palotai, László Halász, Loránd Erőss, Gábor Fekete, László Bognár, Péter Barsi, Andrea Kelemen, Borbála Jávor-Duray, Éva Pichner, Muthuraman Muthuraman, Gertrúd Tamás

**Affiliations:** 1 Department of Neurology, Semmelweis University, Budapest, Hungary; 2 Department of Neurology, Julius-Maximilians-Universität of Würzburg, Würzburg, Germany; 3 Department of Neurosurgery and Neurointervention, Semmelweis University, Budapest, Hungary; 4 Department of Neurosurgery, University of Debrecen, Debrecen, Hungary; 5 Department of Neuroradiology, Medical Imaging Centre, Semmelweis University, Budapest, Hungary; 6 Department of Neurology, Bajcsy-Zsilinszky Hospital and Clinic, Budapest, Hungary; 7 Informatics for Medical Technology, University of Augsburg, Augsburg, Germany

**Keywords:** Parkinson's disease, Parkinson's disease

## Abstract

Compound network dynamics in beta and gamma bands determine the severity of bradykinesia in Parkinson’s disease. We explored its subthalamic stimulation related changes parallel with improvement of complex hand movements. Thirty eight patients with Parkinson’s disease treated with bilateral stimulation accomplished voluntary and traced spiral drawing with their more affected hand on a digital tablet. A 64 channel electroencephalography was recorded, low and high beta and gamma power was computed in subthalamic and motor cortical sources at four stimulation levels. Subthalamic cortical effective connectivity was calculated, and subnetwork models were created. Beta power decreased, and gamma power increased in sources ipsilateral to stimulation with increasing stimulation intensity. Networks comprising the primary motor cortex played a dominant role in predicting the improvement of voluntary drawing speed. Subthalamic stimulation diminished the hyperdirect high beta information processing and promoted the cortico cortical interactions of the primary motor cortex in the high gamma band.

## Introduction

The compound neuronal network dynamics and its relation to improving bradykinesia during bilateral subthalamic deep brain stimulation (STN-DBS) in Parkinson’s disease (PD) have not yet been fully explored^1^. Recognition of nervous system biomarkers representing the severity of symptoms such as bradykinesia is a key step for developing the adaptive DBS^2–4^, which may further increase the quality of life of the operated patients with advanced PD.

Local field potential studies proved a significant correlation between subthalamic beta-band power^5^, burst duration^6^, and the severity of motor symptoms parallel improving after dopaminergic medication in the low beta band (13–20 Hz)^7,8^ or in a narrow beta band with the individual beta peak as center frequency (18–23 Hz)^9^. Moreover, dynamically changing subthalamic-cortical subnetwork oscillations in the beta and gamma bands detected by electroencephalography (EEG) could predict hand movement kinematic parameters in our previous study^10^ with high accuracy, suggesting a multi-sensing method to capture network activity for improving adaptive stimulation.

When testing the network elements, previous studies found that excessive beta band (13–35 Hz) oscillatory activity determines the deficient subthalamic-cortical processes in PD^11–14^. While low beta activity in the STN reacts actively to levodopa intake^15,16^, high beta activity operates along the hyperdirect pathway between the STN and the primary motor cortex (M1)^13,17^, premotor cortex^14^, and supplementary motor area^1,13,14^ and responds to stimulation adjustment^16^. On the other hand, broadband cortical gamma synchronization (35–100 Hz^10,18^, 50–200 Hz^19,20^, or even 300–400 Hz^21^) involving the primary motor cortex is high in Parkinson’s disease^19,20^, suggesting a compensatory role against dopamine deficiency^20^; it enhances during movement^18,20^ and DBS^10^. The narrow band, finely tuned gamma (FTG) activity (60–90 Hz) is coherent in the STN and sensorimotor cortex^21,22^ and increases after levodopa intake^21,23^ or STN stimulation^24^; it increases further with voluntary movements^21^. Investigations of stimulation-related changes in the low and high beta and gamma band are restricted^10^ in networks with extended motor cortex regions.

Concurrently combined testing methods were applied to measure activity from different subcortical and cortical network elements. The perioperative measurements of subthalamic local field potential in combination with implanted electrocorticography (ECoG) strips above restricted motor cortex areas^17^ or EEG^13,23^/magnetoencephalography (MEG)^1,12,14,21,25^ are affected by the stun effect^1^. Subthalamic local field potential was measured through externalized leads in the chronic phase during the replacement of the impulse generator^3,26^ or by chronically implanted sensing stimulators^5^; however, cortical activity during these measurements was not assessed. Indeed, EEG is a safe method and can be used in many patients who are stimulated chronically also with non-sensing impulse generators. In this way, the changes in the subthalamic-cortical connections and the intercortical processes can be mapped in detail.

Previous studies have investigated the neural basis of bradykinesia predominantly through simple motor tasks^3,27,28^. However, modeling everyday movement tasks, and multi-joint movements, such as drawing or handwriting, have been proposed to yield more information about the pathophysiology of the cortico-basal ganglia network in PD^29^.

However, the changes induced by DBS at the network level during complex graphomotor tasks are yet to be described.

In this study, we investigated how the cortico-subcortical network activity of Parkinsonian patients is influenced by adjusting the stimulation intensity during complex, visually controlled, and self-paced hand movements. We aimed to identify motor subnetworks using an EEG-based source estimation technique whose stimulation-dependent activity may predict the improvement of bradykinesia during complex hand movements. We also aimed to explore stimulation- and task-related reactions in frequency-specific connectivity between the subnetwork elements. We assume multiplex activity feature perception of complex network dynamics is required to accurately predict bradykinesia in PD.

## Results

### Clinical characteristics of the patients

Table 1 presents demographic data, disease-related information, and stimulation parameters. The study involved 24 male and 14 female patients, with a mean age of 65.6 ± 7.38 years. Mini-Mental State Examination (MMSE) scores measured during the time of spiral acquisition did not differ from the preoperative MMSE scores (normal distribution, paired *t*-test, *p* > 0.05) similar to the Addenbrooke’s Cognitive Examination scores (normal distribution, paired *t*-test, *p* > 0.05).

**Table 1:** Demographics and clinical data of the recruited patients

Mean age (SD)		65.6 (7.38) years
Sex		14 females, 24 males
Disease duration		13.2 (6.67) years
Elapsed time after operation		2.7 (2.20) years
Left STN stimulation	Amplitude	2.3 (0.59) mA
	Pulse width	59.9 (6.06) us
	Frequency	134.8 (7.36) Hz
Right STN stimulation	Amplitude	2.3 (0.83) mA
	Pulse width	60.7 (7.78) us
	Frequency	135.2 (7.4) Hz
Tested STN		24 left, 14 right
Preoperative UDPRS III.	MED OFF	23.1 (18.46) points
	MED ON	8.3 (9.03) points
UPDRS III. at the time of the study	STIM ON-MED OFF	10.7 (12.14) points
	STIM ON-MED ON	5.2 (6.47) points
Hoehn-Yahr stage	Preoperative	1.7 (0.83)
	At the study	1.3 (0.84)
Levodopa equivalent dose	Preoperative	745.3 (540.38) mg
	At the study	407.4 (281.07) mg
Mini-Mental State Examination scores	Preoperative (38/38 patients)	28.34 (1.26)
	At the study (38/38 patients)	28.32 (0.96)
Addenbrooke’s Cognitive Examination scores	Preoperative (23/38 patients)	89.4 (5.35)
	At the study (35/38 patients)	87.4 (5.79)

*STN* subthalamic nucleus, *UPDRS* Unified Parkinson’s Disease Rating Scale.

**Fig. 1:**
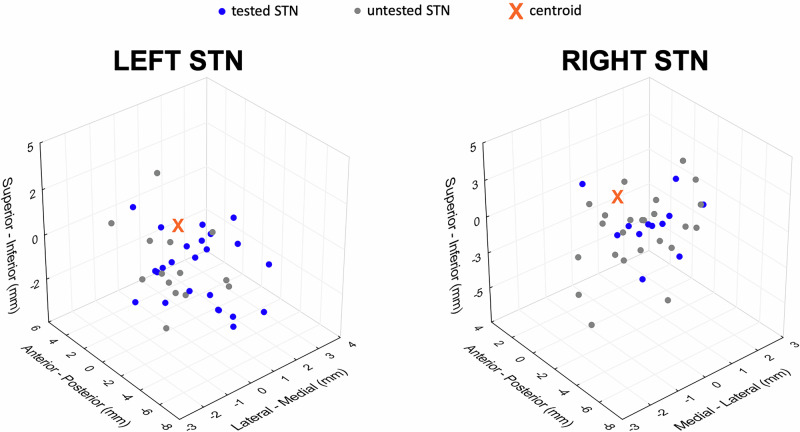
Active contact locations relative to the center point of the dorsolateral STN. The active contact locations are presented in the MNI space; the *x*-axis represents the medial-lateral, the *y*-axis anterior-posterior, and the *z*-axis superior-inferior plane. Tested contact locations in each STN contralateral to the movement are presented with blue dots. Gray dots depict contact locations of untested STN (ipsilateral to the movement) with stable stimulation. The center point of the dorsolateral STN is the point of reference, represented by an orange cross. MNI Montreal Neurological Institute, STN subthalamic nucleus.

Figure 1 displays the position of stimulating contacts within the sensorimotor region of the STN for all patients. The mean distances (±SD) of the tested active contact from the center of the dorsolateral STN were: X: 1.04 ± 0.66 mm; Y: 2.46 ± 1.33 mm; Z: 1.25 ± 1.1 mm; 3D distance: 0.77 ± 0.76 mm.

The patients performed self-paced and traced spiral drawing at four different stimulation levels in randomized order. Subsequently, the intensity (average ± SD) of the various stimulation levels was at level 0: 0 mA, at level 1: 1.25 ± 0.58 mA, at level 2: 2.32 ± 0.73 mA, and at level 3: 2.99 ± 0.84 mA.

### Spiral drawing analysis

**Fig. 2:**
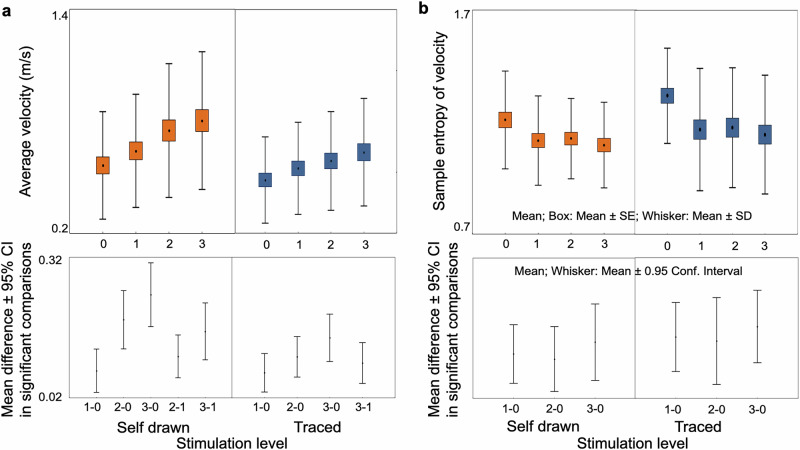
Average velocity and entropy of velocity of spiral drawing in the four levels of stimulation. **a** Average tangential velocity increased in both self-paced and traced spiral drawing tasks. **b** Entropy of velocity decreased when switching the stimulation on but did not follow further intensity increase in each task. Repeated-measures ANOVA revealed a significant main effect of stimulation level. Significant post hoc comparisons are presented in the bottom row (*p* < 0.05).

The average time needed to draw a single spiral was 14.3 ± 6.42 s, while the average time required to draw 5 spirals was 64.7 ± 23.71 s. The average tangential velocity was higher in the self-paced than in the traced drawings (repeated measures ANOVA; TASK factor: *F*_1,37_ = 33.40, *p* < 0.0001). It increased gradually with increasing stimulation intensity during both the self-paced and traced drawing tasks (STIMULATION LEVEL factor: *F*_3,111_ = 27.43, *p* < 0.001; Fig. 2a). There was a significant difference in the slope of increase of the average tangential velocity, being higher in the case of self-paced spirals than traced spirals (non-normal distribution, Wilcoxon matched-pairs signed rank test, *p* = 0.0035). The velocity entropy was lower in the self-paced than in the traced spiral drawing (TASK factor: *F*_1,37_ = 14.95, *p* < 0.0001). In addition, the entropy of velocity in both tasks diminished when turning the stimulation on but did not change parallel with increasing stimulation intensity (STIMULATION LEVEL FACTOR: *F*_3,111_ = 12.74, *p* < 0.001. Tukey significant post hoc comparisons: self-paced and traced drawing: *p*_0-1_ < 0.05, *p*_0-2_ < 0.05, *p*_0-3_ < 0.05; Fig. 2b). The rate of decrease in the entropy of velocity did not differ between the two types of drawing (normal distribution, paired *t*-test, *p* > 0.05).

### Power spectral density analysis in the beta and gamma bands

**Fig. 3:**
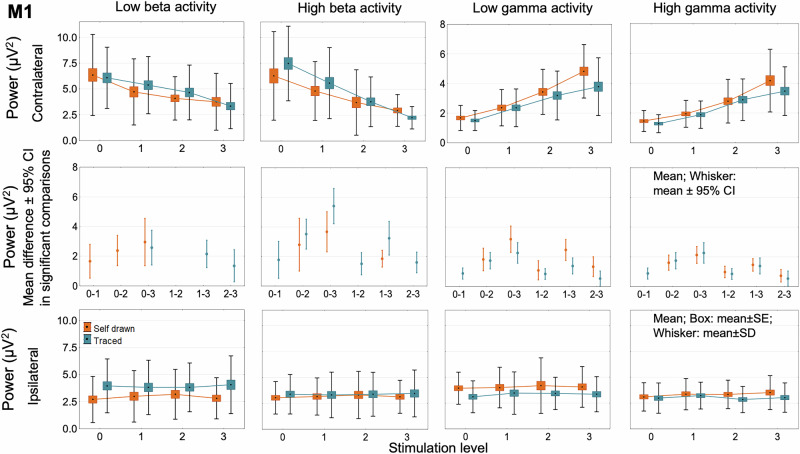
Absolute beta and gamma power changes in the primary motor cortex. In the primary motor cortex, subthalamic stimulation decreased absolute low and high beta band power stepwise in the tested hemisphere (contralateral to the movement). At the same time, low and high gamma activity was raised with increasing stimulation intensity. Power differences in significant post hoc comparisons of stimulation level effect are presented in the middle row. Beta and gamma power remained unchanged in the untested hemisphere, with rising stimulation intensity. Bottom row: in the not-tested hemisphere (ipsilateral to the movement), beta and gamma power remained unchanged with elevating stimulation intensity. M1 primary motor cortex.

**Fig. 4:**
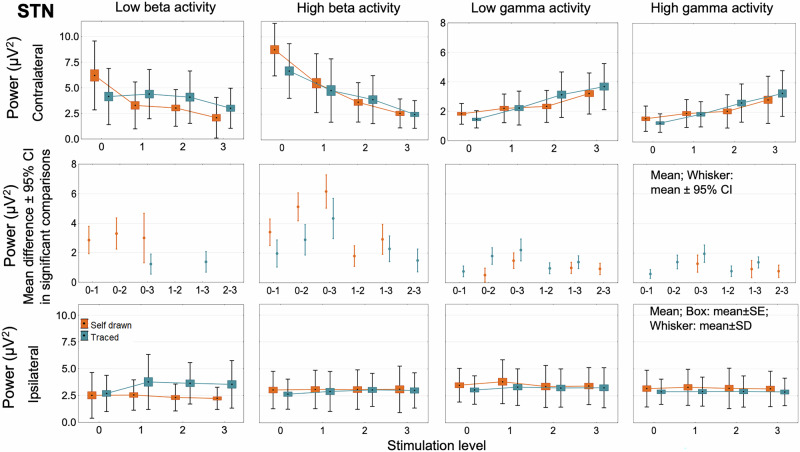
Absolute beta and gamma power changes in the subthalamic nucleus. Stimulation-induced power changes in the low beta, high beta, low gamma, and high gamma bands in the STN are similar to the changes observed in the primary motor cortex (Fig. 3). STN subthalamic nucleus.

During both self-paced and traced spiral drawings, the power spectral analysis of each frequency band revealed significant changes when modifying the DBS setting contralateral to the movement (ipsilateral to the stimulation) in the cortical areas (primary motor cortex: M1, dorsal premotor cortex: DPMC, ventral premotor cortex: VPMC, supplementary motor area: SMA, pre-supplementary motor area: pre-SMA, dorsolateral prefrontal cortex: DLPMC) and the STN (LOC × BAND × SUBBAND × HEMISPHERE × STIM LEVEL within effect interactions: self-paced task: *F*_24,432_ = 1.8, *p* = 0.012; traced task: *F*_24,456_ = 2.4, *p* = 0.0002; Figs. 3 and 4 and Supplementary Figs. 1–6).

As the stimulation intensity increased, we observed a gradual decrease in both low beta and high beta power, accompanied by a gradual increase in low gamma and high gamma power during the graphomotor tasks contralateral to the movement (ipsilateral to the stimulation). Power values in the four frequency bands in the cortical areas and the STN ipsilateral to the movement (contralateral to the stimulation) were not influenced by the intensity changes of the DBS (Figs. 3 and 4 and Supplementary Figs. 1–6).

We have chosen the visual cortex as reference because it is not related to motor actions; neither beta nor gamma band activity changed with the adjusted stimulation intensities (Supplementary Fig. 6).

### Prediction analysis

We examined if we could predict the stimulation-induced improvement (slope of data set at the 0–3 stimulation levels) in self-paced and traced spiral drawing speed from the stimulation-induced low, high beta and gamma power changes (slope of data set at the 0–3 stimulation levels) of the cortical motor sources and the STN.

The optimal model for both self-paced and traced spirals was trained with eight features of EEG spectral power for each frequency band separately. The prediction accuracy of R-squared were (self-paced: *R*^2^ = 0.63 ± 0.1; traced: *R*^2^ = 0.71 ± 0.03). Based on the mean absolute SHapley Additive exPlanations (SHAP) values, we identified the most influential features affecting the support-vector machine (SVM) prediction of stimulation-induced change in self-paced spiral drawing speed. High beta power in the DLPFC, DPMC, and M1 emerged as key factors. High gamma power in the M1 and SMA and the DLPFC, with SHAP values of 0.019, also significantly contributed to deviations from the average prediction of self-paced drawing speed slope across all instances (Supplementary Table 1). The stimulation-induced power slopes of sources in the four frequency bands did not reliably predict the drawing speed slope of traced spirals (Supplementary Table 2).

### Effective connectivity

We investigated how effective connectivity between sources is affected by the stimulation intensity level.

**Fig. 5:**
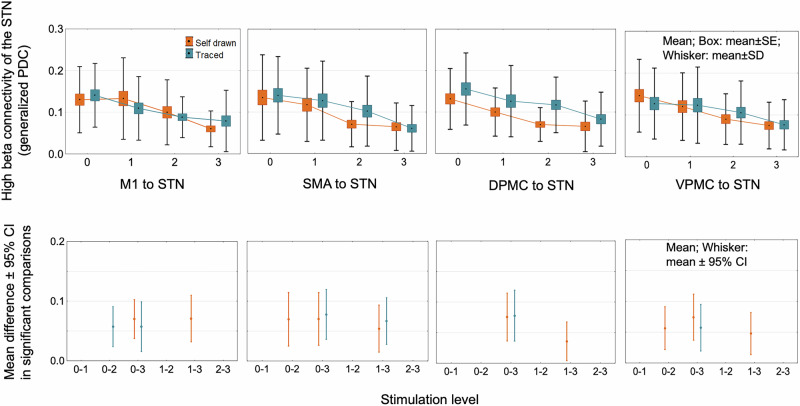
Stimulation effects on task-related hyperdirect high-beta effective connectivity in PD patients. Effective connectivity between the motor cortical areas and STN decreased in the high beta band with increasing stimulation intensity. Significant comparisons of stimulation level effects are presented in the bottom row. The figure visualizes the direction of coherence from motor cortical areas toward the subthalamic nucleus. However, this effect was bidirectional; the opposite directional effective connectivity is demonstrated in Supplementary Fig. 7. DPMC dorsal premotor cortex, M1 primary motor cortex, SMA supplementary motor cortex, STN subthalamic nucleus, PD Parkinson’s disease, VPMC ventral premotor cortex.

**Fig. 6:**
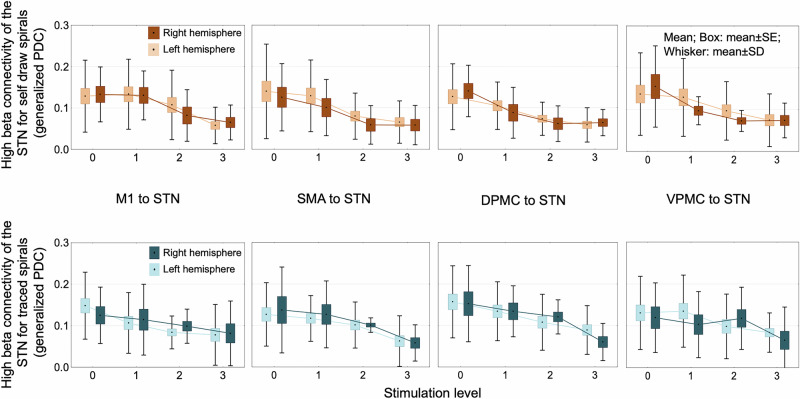
Stimulation effects on task-related hyperdirect high-beta effective connectivity in PD patients according to the tested hemisphere. The comparison of the tested left and tested right hemispheres revealed a similar stimulation effect on connectivity values with the merged analysis (Fig. 5): effective connectivity between the motor cortical areas and STN decreased in the high beta band with increasing stimulation intensity. Effective connectivity in patients with tested left hemisphere did not differ from those with tested right hemisphere in any stimulation level and pair of brain regions (*p* > 0.05). The figure presents the direction of coherence from motor cortical areas toward the subthalamic nucleus. However, this effect was bidirectional; the opposite directional effective connectivity is demonstrated in Supplementary Fig. 8. DPMC dorsal premotor cortex, M1 primary motor cortex, SMA supplementary motor cortex, STN subthalamic nucleus, PD Parkinson’s disease, VPMC ventral premotor cortex.

**Fig. 7:**
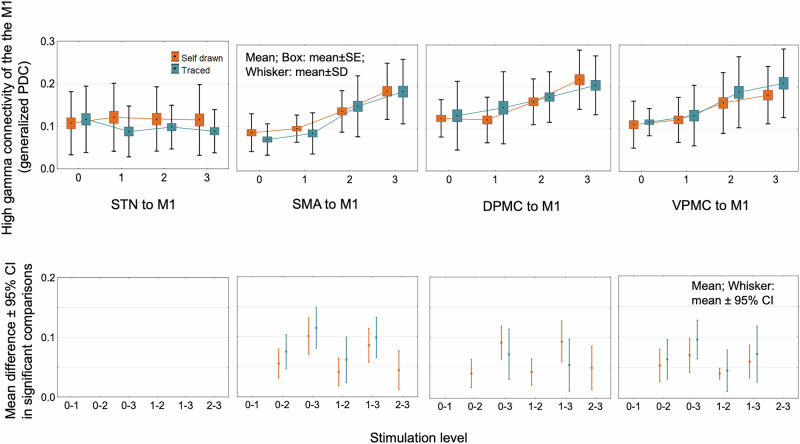
STN stimulation increases the high-gamma effective connectivity between the premotor cortical areas, and the M1. Ramping subthalamic stimulation increased the effective connectivity between the premotor cortical areas and M1 in the high gamma frequency band; however, the high gamma STN-M1 effective connectivity was unaffected. Significant post hoc comparisons of the stimulation level effect are presented in the lower row. The effect was bidirectional; effective connectivity from the direction of M1 to other structures is presented in Supplementary Fig. 9. DPMC dorsal premotor cortex, M1 primary motor cortex, SMA supplementary motor cortex, STN subthalamic nucleus, PD Parkinson’s disease, VPMC ventral premotor cortex.

For both self-paced and traced spiral drawings, the generalized partial directed coherence effective connectivity (EC) in the high beta band decreased with increasing stimulation intensity between the STN and the cortical areas, such as M1, SMA, VPMC, and DPMC contralateral to the movement, and the effect was present on both direction (STN to motor cortex: STIMULATION LEVEL factor: *F*_3,68_ = 84.17, *p* < 0.0001; motor cortex to STN: STIMULATION LEVEL factor: *F*_3,68_ = 19.07, *p* < 0.0001) (post hoc comparisons: Fig. 5 and Supplementary Fig. 7). We compared the tested left hemispheres (24 patients) with the tested right hemispheres (14 patients), and there was no difference in the EC values (TESTED SIDE factor: *F*_1,22_ = 0.34, *p* > 0.05) (post hoc comparisons: Fig. 6 and Supplementary Fig. 8). At the same time, EC increased in the high gamma band between M1 and premotor cortical areas (SMA, VPMC, and DPMC) in both directions (M1 to premotor cortical areas: STIMULATION LEVEL factor: *F*_3,68_ = 49.93, *p* < 0.0001; premotor cortical areas to M1: STIMULATION LEVEL factor: *F*_3,68_ = 62.88, *p* < 0.0001), but not between STN and motor cortical areas (*p* > 0.05 in all post hoc Tukey test comparisons) (Fig. 7 and Supplementary Fig. 9). In the analyzed regions, there was no stimulation-induced change in EC in the low beta and low gamma frequency bands (*p* > 0.05 in all comparisons). There was no significant difference in connectivity values measured during traced and self-paced drawings (TASK factor: *F*_1,15_ = 0.029, *p* > 0.05).

## Discussion

This study explored the frequency-specific network effects of subthalamic nucleus stimulation during complex hand movements in patients with Parkinson’s disease. First, we showed that STN DBS suppressed the synchronized high beta band oscillatory activity between the STN and motor cortical areas while facilitating the high gamma information flow between the motor cortex areas in a dose-dependent manner in both tasks. Second, we showed that the speed of spiral drawing increased with increasing stimulation intensity. At the same time, low and high beta power and low and high gamma power gradually decreased and increased, respectively, throughout the cortico-subthalamic motor network during traced and self-paced spiral drawing. Third, we also show that high beta and high gamma oscillation in networks, including M1, most accurately predicts speed improvement in self-paced spiral drawing. What sets our study apart from previous findings in the literature is the assessment of STN stimulation-induced changes in the cortico-subthalamic connectivity profile during fine motor control under different stimulation intensities.

With our source reconstruction model, we could show that subthalamic stimulation reduces connectivity between the STN and cortical regions, including the M1, SMA, DPMC, and VPMC at the high beta (21–30 Hz) frequency range. STN-DBS did not influence cortico-subthalamic effective connectivity in the low beta and low gamma frequency ranges.

The hyperdirect pathway has a crucial role in developing bradykinesia^30^, and its subthalamic entry point represents the sweet spot for effective subthalamic deep brain stimulation targeting in PD^31,32^.

An fMRI measurement has shown increased functional connectivity along the hyperdirect pathway even in the early stages of PD^33^, with a contralateral preponderance to the more affected limb in the resting state^33^. This phenomenon was decreased at rest and increased during movement by STN-DBS, which was associated with better motor performance^34^.

In STN local field potential (LFP) measurements combined with ECoG^17^, EEG^35,36^, or MEG^1,14,37^, it was shown that the high beta frequency connectivity is dominant along the hyperdirect pathway and decreases with STN stimulation^1,14^, which is consistent with our results. It was hypothesized that high beta activity in the hyperdirect pathway enhances subthalamic low beta oscillations, which correlate most with the severity of bradykinesia^1,14^.

Pathological high beta subthalamo-cortical coherence was also revealed in animal models of Parkinson’s disease^38^. It is up for debate whether the cortex^1,14,17,35,36^ drives the STN or if the connectivity between them is bidirectional^13,39^. An LFP study combined with MRI supposed that decreasing cortical thickness may play a role in the emergence of pathological high beta activity^40^. According to our results, the effective connectivity in the high beta band along the hyperdirect pathway diminishes gradually with increasing subthalamic stimulation in both directions. However, it has also been described that the effective direction of coherent oscillatory activity in the basal ganglia is dynamic and depends on the brain state^41^.

Earlier studies also found that STN activity at 60–90 Hz was coherent with activity in M1; directionality analysis showed that STN gamma activity at 60–90 Hz tended to drive gamma activity in M1^21^. In this study, we could not explore stimulation-reactive hyperdirect activity in the gamma bands. The hyperdirect pathway activity was shown to be lateralized. Functional MRI data of healthy subjects revealed that effective connectivity of the right hyperdirect and direct pathways could predict the best outcome of the stop-signal task^42^. Furthermore, the lateralized activity of the STN was indicated by a resting state STN LFP study of 24 patients with Parkinson’s disease, which revealed that phase-amplitude coupling between high beta (20–30 Hz) and high gamma (70–100 Hz) oscillations is higher in the right STN than in the left STN irrespective to the asymmetry in pathophysiology, in the off-levodopa condition and reacts better to levodopa intake^43^. In our study of 38 patients, we found that the subthalamo-cortical effective connectivity did not differ significantly in more affected and tested left and right hemispheres. Here, we assessed the coherent activity between the two distinct regions, and the mentioned study analyzed cross-frequency coupling (CFC) inside the STN. Further studies are needed to unravel the role of CFC along the left and right hyperdirect pathways in patients treated with deep brain stimulation.

At the cortical level, subthalamic stimulation improved bidirectional effective connectivity between M1 and other motor cortical areas, such as the SMA, VPMC, and DPMC, parallel with the clinical improvement. Still, this phenomenon was absent between the SMA and the premotor cortical areas.

Cortical dysfunction is also part of the compound network dysfunction related to bradykinesia in PD. In the resting state, pre-SMA showed deficient connectivity exposed by fMRI with surrounding cortical areas, including the ipsilateral premotor cortex, insula, and parietal cortex, and increased connectivity between pre-SMA and M1^44^. Earlier studies scrutinized the lower gamma band connectivity at the cortex network level. A previous EEG study found an increased widespread cortical low-gamma (30–45 Hz) synchrony in de novo PD patients^45^, and the authors argued that it might be a transient compensatory mechanism of preserved cortical neurons and represents a motor capacity reactive to levodopa^21^ and STN stimulation^46^. It was shown by EEG that augmented cortical gamma (35–50 Hz) connectivity of the central cortical areas promotes movement initiation and execution^45^. Globus pallidus internus and subthalamic nucleus DBS increased resting state synchrony in the 26 to 50 Hz frequency band between the prefrontal, motor, middle/inferior temporal, and occipitoparietal cortices in a MEG study^47^.

It is unclear how the high gamma band power and connectivity subserve motor cortical projections and how STN-DBS supports them. The effects of ramping STN stimulation on the interaction of motor cortical regions have not been assessed in the chronic postoperative phase and during fine motor control.

STN-LFP and MEG analysis restricted to the M1 yielded high gamma band (60–90 Hz and 300–400 Hz) peaks increasing with movement and levodopa therapy^21^. STN-M1 coherence was also identified in this study in the finely-tuned gamma band (60–90 Hz); it was negatively correlated with clinical symptoms and was driven by the STN^21^. We note that STN-DBS did not affect the subthalamo-cortical connectivity in the high gamma band (60–100 Hz) comprising the finely-tuned gamma band in our study. A possible mechanism of action of subthalamic stimulation might be that it hampers the pathological beta-driven network inhibition and activates the cortex antidromically through the hyperdirect pathway^48^. Our results support this hypothesis as we observed parallel reduction in the high beta hyperdirect connectivity and enhancements of high gamma information processing between the M1 and other motor cortical areas.

Spiral drawing is a complex hand movement that has been less studied concerning the mechanism of action of deep brain stimulation^49,50^. However, analyzing complex movements requiring different degrees of continuous sensory feedback can bring us closer to modeling everyday activities. In Parkinsonian patients, the quantitative analysis of spiral drawings previously showed that linear parameters, including drawing speed, are markers of disease severity^51,52^, and correlate with the motor scores of the Movement Disorder Society-Unified Parkinson’s Disease Rating Scale (MDS-UPDRS-III)^51^. It may discern early signs of Parkinson’s disease with high sensitivity^51^. Moreover, spiral testing can objectively monitor the beneficial impact of STN stimulation in the early postoperative phase^49,50^ and reacts to medication state^53^.

In this study, tangential velocity increased with ramping stimulation intensity in traced and self-paced spiral drawings. Nonetheless, the entropy of velocity in both tasks only reacted to switching the stimulation on, but it did not follow the gradual increase in stimulation intensity. Dopaminergic substitution^54^ and subthalamic stimulation^10,55^ predominantly improved the speed of repetitive hand movements over the amplitude in former studies. The rhythm reacted the least, and the decrement of speed and amplitude was resistant to both therapy^10,54,55^, suggesting irregularities in time and space in accord with our present observation of limited stimulation-induced entropy decrease.

During both self-paced and traced spiral drawings, ramping STN-DBS decreased low and high beta band power and increased low and high gamma band activity in the motor cortical areas and the subthalamic nucleus in a dose-dependent manner. As a reference, we did not find stimulation-induced beta and gamma power changes in the visual cortex from either side. We experienced these apparent changes only in the tested hemisphere while the other sided stimulation was continuously set on the therapeutic level similarly to our previous observations with repetitive hand movements^10^. These results support the observation that the cortico-subthalamic pathway is strictly ipsilateral^56^. We also report that the slope of high beta activity of a subnetwork comprising of DLPFC, DPMC, and M1 and the high gamma activity of another subnetwork involving the SMA and M1 predicted the slope of self-paced but not the traced spiral drawing velocity during ramping stimulation. It indicates that high beta and high gamma oscillation in networks, including M1, most accurately predicts speed improvement in self-paced spiral drawing. We could not achieve a similar prediction for the traced spiral drawing task, probably because of the additional involvement of the visual and association cortex areas in the motor action.

In single-element STN LFP recordings, beta-band activity was dominant in the sensorimotor part of the STN with inputs from the primary motor cortex^57^. The decrease in low beta activity correlated with the alleviation of the bradykinesia in the early^2,7^ and chronic^5^ postoperative phase. Tinkhauser et al. demonstrated that intraoperative beta band power suppression induced by directional STN-DBS predicted treatment efficacy^58^, and it was hypothesized that motor function preservation may rely on the modulation of subthalamic beta LFP activity^59^. Prolonged bursting synchronizations of beta oscillation in the subthalamic nucleus correspond to transient yet excessive increases in beta amplitude, and they have been linked with impaired motor command and execution in PD^60^. The pre-movement timing of subthalamic beta bursts affects movement velocity^61^, and within movement occurrence of beta bursts is associated with speed reduction during self-paced spiral drawing but not during cued fine motor movements^49^. Even the intraburst rate of spikes positively correlated with bradykinesia and axial scores but not tremor^46^. Bradykinetic symptom alleviation by dopaminergic medication was related to a reduction in beta burst duration in the 13–20 Hz range^7^, however, the effect of conventional STN DBS on subthalamic beta burst dynamics remain inconsistent. Intraoperative STN DBS of awake patients did not influence burst rate and duration at rest^62^ while ramping stimulation during repetitive hand movements led to attenuation in beta burst duration^9^ and burst amplitude decreased during fine motor control^49^ in chronically implanted patients. To clarify the relation of beta burst to effective stimulation, comprehensive experimental protocols are needed taking into account that burst duration, amplitude, and rate are more pronounced in the resting state^49^.

Our results pinpointed that the frequency^10,37^, and task-specific^10^ subnetwork activity predicts bradykinesia severity, in which the M1 and the STN play crucial role and multisensing technique may point the way forward for the development of adaptive stimulation.

Our results highlight that specific changes in the cortical and subthalamic connections of the primary motor cortex determine the effectiveness of stimulation. Preoperative examination of high gamma effective connectivity between the primary motor cortex and other motor cortical areas and the high beta hyperdirect connectivity under a levodopa challenge test using EEG might have predictive value for individual surgical outcomes. Studies using machine learning could explore these functional and structural network features further.

The study is limited by the disproportionate distribution of male and female patients and the lack of healthy controls. However, the relatively large number of patients included ensures a reliable representation of the statistical sample. The different spirals, namely the traced and self-paced spirals, were drawn only 5 times at each stimulation level. In the future, more trials might further increase the robustness of statistics. As mentioned earlier, the region of interest analyses pipeline in this study included subcortical sources with 64-channel EEG data. To optimize the spatial filter source signals, we used the individual T1 and the FEM models. We have selected those frequency bands, namely beta and gamma, with higher signal-to-noise ratio during a motor task with visual feedback to get the optimal source signals.

In conclusion, our study indicates that subthalamic stimulation-reactive high beta hyperdirect activity and high gamma M1-related cortical connections determine the change of bradykinesia during ramping the intensity; this outlines a need for a complex multisensing method targeting the subthalamic and motor cortex locations to capture symptom-related biomarkers in Parkinson’s disease^4^.

## Methods

### Study participants and protocol

**Fig. 8:**
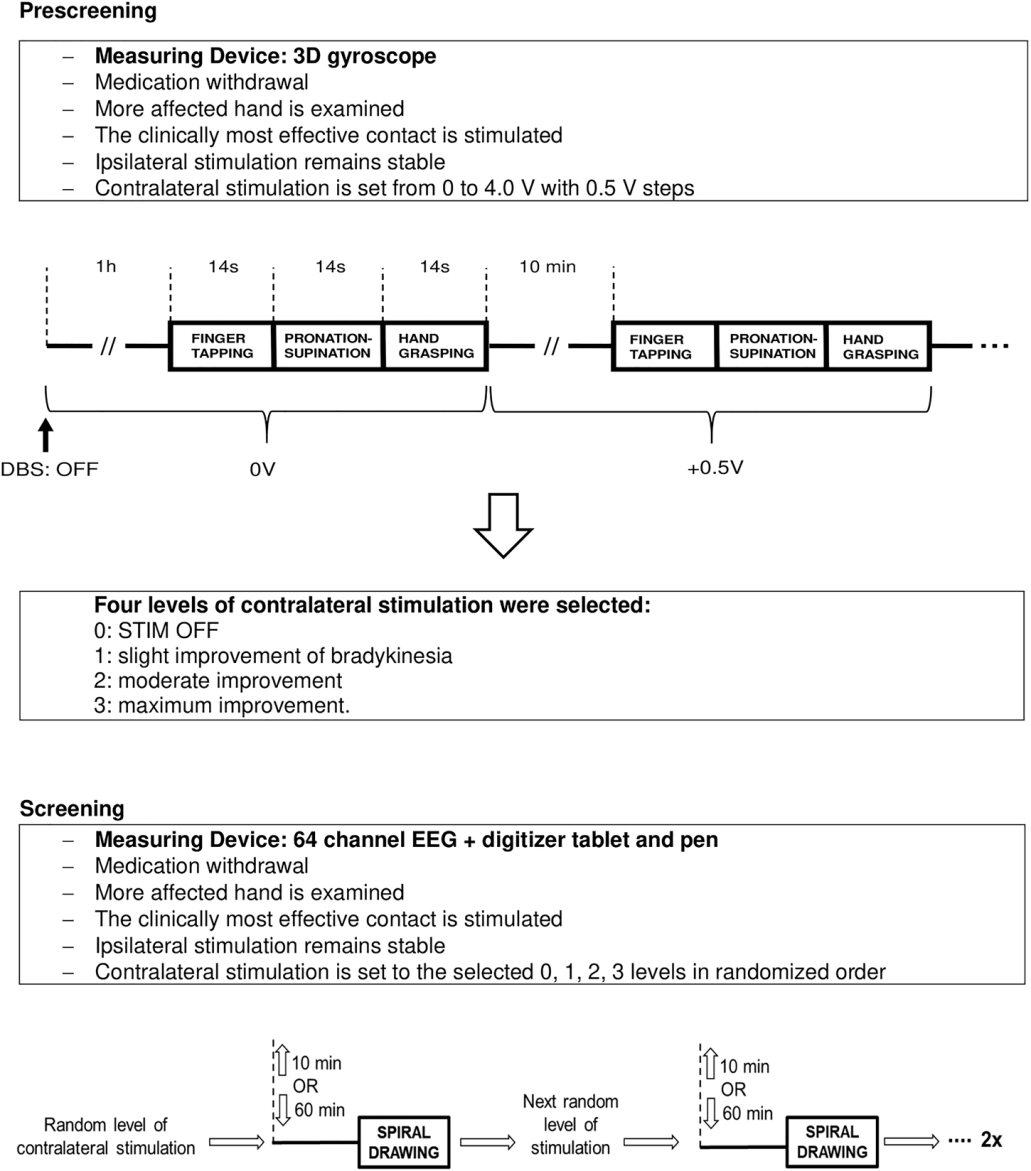
Study protocol. EEG electroencephalography, STIM OFF stimulation OFF.

Thirty-eight patients diagnosed with idiopathic Parkinson’s Disease and treated with bilateral STN-DBS for at least 1 year were recruited at the Department of Neurology, Semmelweis University, Hungary. The patients signed an informed consent form according to the Declaration of Helsinki. The Medical Research Council in Hungary has provided ethical approval (080958/2015/OTIG) for the study. Inclusion criteria were bradykinesia and rigidity as leading symptoms. Candidates previously diagnosed with musculoskeletal disorders or dementia were excluded from the study. Motor performance was assessed using the Hoehn and Yahr Scale and the Unified Parkinson’s Disease Rating Scale Part III (UPDRS-III). We converted the MDS-UPDRS III. preoperative scores in 2/38 patients and the postoperative scores in 10/38 patients to the scores of the UPDRS III. scores^63^. The dopamine agonists were only stopped 1 day before the test because patients did not tolerate the discomfort. The study protocol is summarized in Fig. 8.

### Surgical procedure

Bilateral DBS leads (in 23 patients: Medtronic 3389; 9 patients: St Jude 6147; 7 patients Abbott 6170; 1 patient: Boston 2202) were implanted using standard stereotactic procedures^64^ with the aid of microelectrode recording and clinical testing. Impulse generators were connected on the same day. A detailed description of the surgical procedure has been previously published^55^. The patients had no surgical or hardware-related complications in the perioperative phase.

### DBS electrode localization

The DBS electrode localization of all patients was determined using the default settings of an advanced processing pipeline in the Lead-DBS software, version 3.1 (https://www.lead-dbs.org/)^65^. Our approach involved linear co-registrations of post-operative head CT or MRI scans to pre-operative T1-weighted images using Advanced Normalization Tools (ANTs; http://stnava.github.io/ANTs/)^66^. Co-registration results were corrected for potential intra-operative brain shift using an automated subcortical refinement module within Lead-DBS. We used the STN atlas definitions from the DBS Intrinsic Template (DISTAL) atlas, version 1.1^67^, for all analyses and visualizations. This precise subcortical atlas was explicitly created for use within Lead-DBS and is based on multi-modal MRI, histology, and structural connectivity information. Normalization warp fields were manually refined using the WarpDrive toolbox^68^ included in Lead-DBS, version 3.1^15^, to enhance registration accuracy further. Special attention was given to any visible mismatches in the registration, particularly focusing on the STN as the anatomical structure of interest. The integration of patient-specific active electrode contacts with corresponding stimulation parameters was simulated using an adaptation of the SimBio/FieldTrip pipeline (https://www.mrt.uni-jena.de/simbio/ and http://fieldtriptoolbox.org/)^69^, as implemented in Lead-DBS, version 3.1^65^. Finally, we computed the Euclidean distance between the active contact points and the center of the STN motor segment.

### Prescreening

A prescreening with Kinesia motion capture system (Great Lakes Neurotechnologies Inc., USA) assessed the kinematic parameters of finger tapping, hand grasping, and pronation-supination of the most affected hand the day before the primary measurement. The contralateral DBS lead was turned off for 1 h, and the patients performed each task for 15 s and then repeated the tasks after elevation of stimulation intensity with 0.5 V/mA steps until the level produced the best improvement without provoking side effects. Kinesia speed, amplitude, and rhythm subscores were calculated for each patient and on each stimulation level^54,70^. We determined the total Kinesia scores (0–4) out of the subscores by combining the three hand movement tasks^71^. Using the total Kinesia scores, we selected four distinct levels of contralateral stimulation: 0 for the OFF state, 1 (mild improvement), 2 (moderate improvement), and 3 (maximum improvement without side effects). The clinically most effective contact location was used for test stimulation throughout the study when the patients were on 12-h medication withdrawal. We applied the clinically most effective stimulation configuration, which was unipolar in all patients. Stimulation intensity in Volt was converted to current intensity (mA) according to the individual impedance values.

### Primary measurement, spiral acquisition, and analyses

The day following the prescreening, we performed the primary measurement after a 12-h medication withdrawal. Patients were comfortably seated in front of a table and asked to draw Archimedean spirals with the most affected hand five times with a template (traced) and five times voluntarily (without a template, self-paced) on the four previously selected stimulation levels. We used a wireless inking pen and a digitizer tablet placed on the table [Wacom Bamboo Fun Pen and Touch Small Tablet CTH 461, Wacom Technology Corporation, Vancouver, WA, with a resolution of 2540 lpi (1000 points/cm), a sample frequency of 100 Hz, a pen active area of 14.72 × 9.20 cm and a pressure sensitivity of 1024 levels]. First, subjects were asked to trace a template Archimedean spiral (spacing = 1.5 cm, maximal radius covered by the active zone of the tablet = 5.0 cm, three loops) printed on a sheet of paper (DIN A4 format = 24.0 × 29.7 cm) and attached to the surface of the tablet. Second, patients were asked to freely draw a spiral on the tablet without spatial constraints (i.e., without a template). At the beginning of every session, the patients were instructed to sit with their shoulders parallel to the front edge of the tablet and to draw at their own speed, starting from the center and ending at the border of the active zone without lifting the pen in between. In the case of traced drawing, the border of the active zone was marked on the template; patients were also asked not to cross the lines of the printed spiral. Before data collection, participants were allowed to practice. Spirals were drawn clockwise (right hand) or counterclockwise (left hand) depending on the more affected hand. Sessions of voluntary spiral drawing followed the traced drawing in every patient. The four selected stimulation levels were applied in counterbalanced order. The pertinence of the drawings was checked right after the sessions, and the patients drew additional spirals when it was necessary to reach the sum of 5 appropriate spirals for a given condition and stimulation setting.

The digital tablet was connected via standard USB to a computer. Kinematic data points were recorded using the Neuroglyphics software (http://www.neuroglyphics.org)^72^, a Microsoft-based Windows application developed for kinematic information recording and analysis. The movement onset and end were visually checked and marked offline.

Based on the instantaneous X and Y coordinates, the software determined the first, second, and third derivatives of position, respectively (Supplementary Fig. 10). To accurately characterize the motion of the pen along the circular path, we extracted the tangential velocity calculated for every data point. For this, the software estimated the rate of change of the angular displacement that was multiplied by the instantaneous radius of the spiral. Tangential velocity can be defined as the linear velocity of an object moving along a circular path, measured at an arbitrary instant. Hereupon, we calculated the average and peak values of tangential velocity along with the sample entropy of tangential velocity for every spiral drawing. Sample entropy measured the average uncertainty of a random variable and was estimated with the MATLAB software (The Mathworks, version R2018b, Natick, Ma, USA) function SampEn (Kijoon Lee, 2023; https://www.mathworks.com/matlabcentral/fileexchange/35784-sample-entropyhttps://www.mathworks.com/matlabcentral/fileexchange/35784-sample-entropy). In the case of time series, the sample entropy can be defined as the negative of the natural logarithm of the probability that two similar data sets with the length *m* remain similar if one more data point is added to each of them. We set the length of sequences to *m* = 2. The boundary of similarity above, in which two data sets are considered different, is represented by *r*. It varies between 0 and 1, and in our case, *r* was set to be 20% of the time-series SD. The higher the entropy value was, the higher irregularity was characteristic of a time series. Parameters of five spirals at each stimulation level were averaged for further statistical analysis.

### EEG acquisition and preprocessing

We recorded an EEG file while patients drew five spirals voluntarily and another file while they drew traced spirals under one stimulation condition. These two files were recorded separately under each stimulation condition (4 × 2 files for each patient). The following analysis steps were run on these files separately. Signals were recorded using a 64-channel EEG system (BrainVision Recorder, Brain Products Co., Munich, Germany), sampled at 2500 Hz (Supplementary Fig. 11). The initial preprocessing steps were performed by researchers who were blinded to the stimulation conditions. Preprocessing was carried out using MATLAB and BrainVision Analyzer (Brain Products Co., Munich, Germany). In the first step, data were re-referenced to the common grand average reference of all EEG channels. Then, data were filtered with a fourth-order Butterworth filter: 0.5 Hz high-pass, 300 Hz low-pass, followed by a notch filter: 50, 100, and 150 Hz. In the next step, data were subjected to independent component analyses (ICA and inverse ICA) to remove components related to DBS (Supplementary Fig. 12), muscle, eye blink, eye movement, and line noise artifacts. On average, 6 of 64 components [6.19 ± 1.17 (mean ± standard deviation—SD), were rejected (DBS artifact: 2 ± 2.21; eye artifact: 1 ± 1.05; line noise: 2 ± 0.74; muscle artifacts: 1 ± 0.31)]. Residual muscle artifacts were visually inspected, removed, and interpolated with the cubic interpolation method. In the last step, EEG data recorded during the drawing of five spirals at a given stimulation level were concatenated for further statistical analysis.

### EEG source reconstruction

**Fig. 9:**
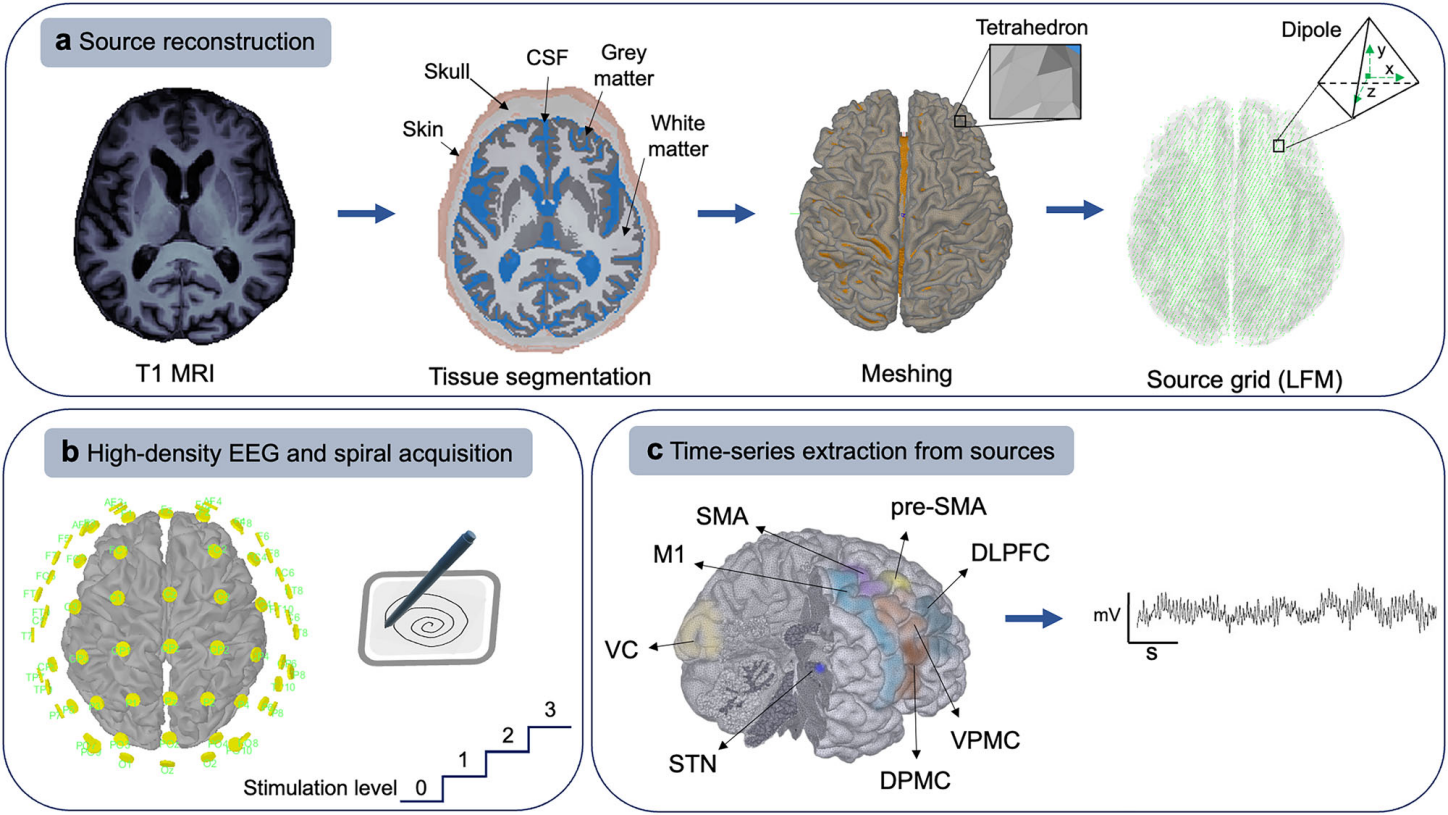
Signal processing pipeline. **a** Based on individual T1 MRI sequences, we reconstructed a lead-field matrix using the finite-element method. **b** High-density EEG acquisition during traced and self-paced spiral drawing at four different stimulation levels. **c** Estimation of coherent neural activity and source extraction from the selected regions. CSF cerebrospinal fluid, DLPFC dorsolateral prefrontal cortex, DPMC dorsal premotor cortex, EEG electroencephalography, LFM lead-field matrix, MRI magnetic resonance imaging, M1 primary motor cortex, SMA supplementary motor cortex, STN subthalamic nucleus, VC visual cortex, VPMC ventral premotor cortex.

To reconstruct the scalp potentials for a set of neural current sources, we modeled the propagation of neural electric currents that produce differences in electrical potentials measured by sensors at the surface (Fig. 9). For this, we used the Brainstorm software^73^ and applied the forward solution with a finite-element method (FEM) which estimated a lead-field matrix (LFM) for the brain^74^. The LFM maps the contribution of a given source activation to potentials at different sensor locations. We proceeded with the following steps for every T1-MRI scan and individual electrode location. (1) A non-linear normalization of the MRIs was made according to the MNI stereotaxic coordinates implemented in the Statistical Parametric Mapping (SPM12) plugin^75^. (2) The CAT12 plugin was used for cortical surface segmentation. (3) The segmentation of the head volume in different tissues (white matter—WM, gray matter—GM, cerebrospinal fluid—CSF, skull, and scalp), represented as high-quality tetrahedral 3D meshes, was achieved with SimNIBS^76^. (4) The head model was generated with DUNEuro based on the individual T1 images and the tetrahedral mesh using a continuous Galerkin solver type and venant source model with a 5 mm grid resolution^77^. For the FEM layers, we assumed isotropic conductivity and used standard conductivity values for each: 0.14 S/m for WM, 0.33 S/m for GM, 1.79 S/m for CSF, 0.008 S/m for skull and 0.43 S/m for scalp. (5) Source computation for each recording and individual electrode location was performed with a linearly constrained minimum variance (LCMV) beamforming method. LCMV is a spatial filtering procedure that monitors the neural activity specified in the head model and estimates their relative contribution to sensor data by passing the electrical activity from a given location while attenuating contributions from other locations and does it for the whole brain so a map of neural power can be created. A detailed description of the inverse solution based on LCMV spatial filtering is given elsewhere^78^. For LCMV beamformers, data noise and signal covariance matrices were estimated first, directly from the recordings. The estimated neural activity was used in further processing. We extracted the relevant motor areas involved in the spiral drawing using a custom-built atlas, namely seven cortical areas (primary motor, dorsal and ventral premotor cortices, supplementary motor area, pre-supplementary motor area, dorsolateral prefrontal cortex, and visual cortex) and the subthalamic nucleus.

### EEG analyses

#### Power spectral density analysis

To perform spectral analysis, A Welch’s periodogram (50% overlap between 1-s Hamming windowed segments) was applied to the artifact-free electroencephalographic signal. The length of the concatenated epochs was 64.7 ± 23.7 s. The power was computed for 4 different frequency bands: low beta (13–20 Hz), high beta (21–30 Hz)^8^, low gamma (31–60 Hz), and high gamma (61–100 Hz)^79^ bands at each of the eight regions separately. We calculated the power values for both contralateral and ipsilateral to drawing of self-paced and traced spirals.

#### Generalized partial directed coherence analysis

We utilized the generalized partial directed coherence (gPDC) method^80^ to determine the directed connectivity between the M1, SMA, DPMC, VPMC, and STN. The gPDC method quantifies the frequency domain direct causal relation using the transformed multivariate autoregressive coefficients A(*f*) in the frequency domain. This method considers the variance (σ) of the original signal and introduces a normalization factor in the denominator. This results in a normalized quantity ($\pi$) describing the ratio of outflow from the region *j* to *i* in relation to all outflows from region *j* (1):

1 $$\left|{\pi }_{{ij}}(f)\right|=\frac{\frac{1}{{\sigma }_{i}}\left|{\bar{A}}_{{ij}}(f)\right|}{\sqrt{\sum _{k=1}\frac{1}{{\sigma }_{k}^{2}}{\left|{\bar{A}}_{k,j}(f)\right|}^{2}}}$$

We estimated the gPDC for the low beta, high beta, low gamma, and high gamma frequency ranges.

### Prediction analysis

In this work, we applied SVM models with Gaussian kernel^81^ on the source power features derived from eight regions of interest, namely (M1, SMA, pre-SMA, DPMC, VPMC, STN, DLPFC, VC) to predict the slope of the spiral velocity computed from the spirals drawn at four different stimulation amplitude. In this work, the SVM models were carried out using the Statistics and Machine Learning Toolbox in MATLAB (R2019a; MathWorks, Natick, Massachusetts, United States of America) with default kernel coefficients and regularization parameters. The complete description of default fitting parameters can be found in MATLAB (Mathworks. Fit a support vector machine regression model. https://de.mathworks.com/help/releases/R2019a/stats/fitrsvm.html, 2019). In this study, SVM models were trained on traced spirals’ source power (eight slope values from eight ROIs) and self-paced spiral source power values. To determine the impact and the contribution of each source power feature from each region on the predicted output of SVM, we used the Shapley value based explanation known as Kernel SHapley Additive exPlanations (SHAP)^82^.

### Statistical analyses

Data analysis was performed using the statistical software TIBCO Statistica version 14.0.1. The normal distribution of the data was checked using the Kolmogorov–Smirnov test. Descriptive statistics were given according to the distribution of the data. To compare the rate of change in tangential velocity and sample entropy across the different levels of stimulation, we extracted the slope of each parameter and compared them using the paired *t*-test and Wilcoxon matched-pairs signed rank test. We compared the average tangential velocity and the entropy of tangential velocity separately at the four stimulation levels in each task. We also analyzed beta and gamma power spectral densities and connectivity strength at the four stimulation levels. Repeated measures analysis of variance (ANOVA) was used with the post hoc Tukey test to detect possible differences in spiral parameters, power spectra, and connectivity strength between different stimulation levels. For the graphomotor tasks, the within-group effects were TASK (self-paced and traced spiral drawing) and STIMULATION LEVEL (0–3). In the case of power spectral density analysis, we used the following within-group effects: TASK (self-paced and traced spiral drawing), BAND (low beta, high beta, low gamma, and high gamma), REGION (M1, DLPFC, VPMC, DPMC, pre-SMA, SMA, VC, STN), HEMISPHERE (contralateral and ipsilateral to the task), STIMULATION LEVEL (0–3). For connectivity analysis, we analyzed the TESTED SIDE (left and right) as between group effect, the within-group effects were as follows: TASK (self-paced and traced spiral drawing), BAND (low beta, high beta, low gamma, and high gamma), PAIR OF REGIONS between M1, SMA, VPMC, DPMC, and STN, STIMULATION LEVEL (0–3). The level of significance was set to *p* < 0.05.

## Supplementary information

Supplementary material

## Data Availability

The dataset for this study can be requested from the corresponding author by qualified researchers, in accordance with the limitations set by the informed consent.
