# Supplementary material for: Subthalamic stimulation evokes hyperdirect high beta interruption and cortical high gamma entrainment in Parkinson’s disease
Source: NPJ Parkinsons Dis. 2025 Apr 26;11:95. doi: 10.1038/s41531-025-00965-6 (PMC12033315; doi:10.1038/s41531-025-00965-6)
Supplement: Supplementary file 1 — Supplementary material [file 41531_2025_965_MOESM1_ESM.pdf]

Supplementary material
Article:
Berki AJ et al. Subthalamic stimulation evokes hyperdirect high beta interruption and cortical high gamma entrainment in Parkinson's disease

ROI	M1	SMA	pre-SMA	DPMC	VPMC	STN	DLPFC	VC
Low Beta	0.0091	0.00905	0.00905	0.0089	0.0089	0.0091	0.00905	0.0089
High Beta	**0.0193**	0.0090	0.0090	**0.0192**	0.0090	0.0090	**0.02**	0.0090
Low Gamma	0.00905	0.0091	0.0089	0.0089	0.0089	0.0089	0.0088	0.0088
High Gamma	**0.019**	**0.019**	0.0085	0.0086	0.0086	0.0085	**0.019**	0.0085

DLPFC: dorsolateral prefrontal cortex; DPMC: dorsal premotor cortex; M1: primary motor cortex; pre-SMA: pre-supplementary motor area; SMA: supplementary motor area; STN: subthalamic nucleus; VC: visual cortex; VPMC: ventral premotor cortex

Supplementary Table 1. Shapley Values for all regions of interest (ROIs) and analyzed frequency bands in self-paced spiral drawings Stimulation-induced M1-related high beta and high gamma network activity changes predicted the parallel increase in drawing velocity

ROI	M1	SMA	pre-SMA	DPMC	VPMC	STN	DLPFC	VC
Low Beta	0.0087	0.0086	0.0085	0.0089	0.0088	0.0093	0.0089	0.0088
High Beta	0.0088	0.0087	0.0086	0.0090	0.00905	0.0089	0.0089	0.0088
Low Gamma	0.00905	0.0090	0.00905	0.0089	0.0090	0.0090	0.0088	0.0091
High Gamma	0.0090	0.0088	0.0085	0.0087	0.0088	0.0086	0.0095	0.0088

DLPFC: dorsolateral prefrontal cortex; DPMC: dorsal premotor cortex; M1: primary motor cortex; pre-SMA: pre-supplementary motor area; SMA: supplementary motor area; STN: subthalamic nucleus; VC: visual cortex; VPMC: ventral premotor cortex

Supplementary Table 2. Shapley Values for all regions of interest (ROIs) and analyzed frequency bands in traced spiral drawings We could not detect any stimulation-induced network activity, which could predict the traced spiral drawing velocity changes

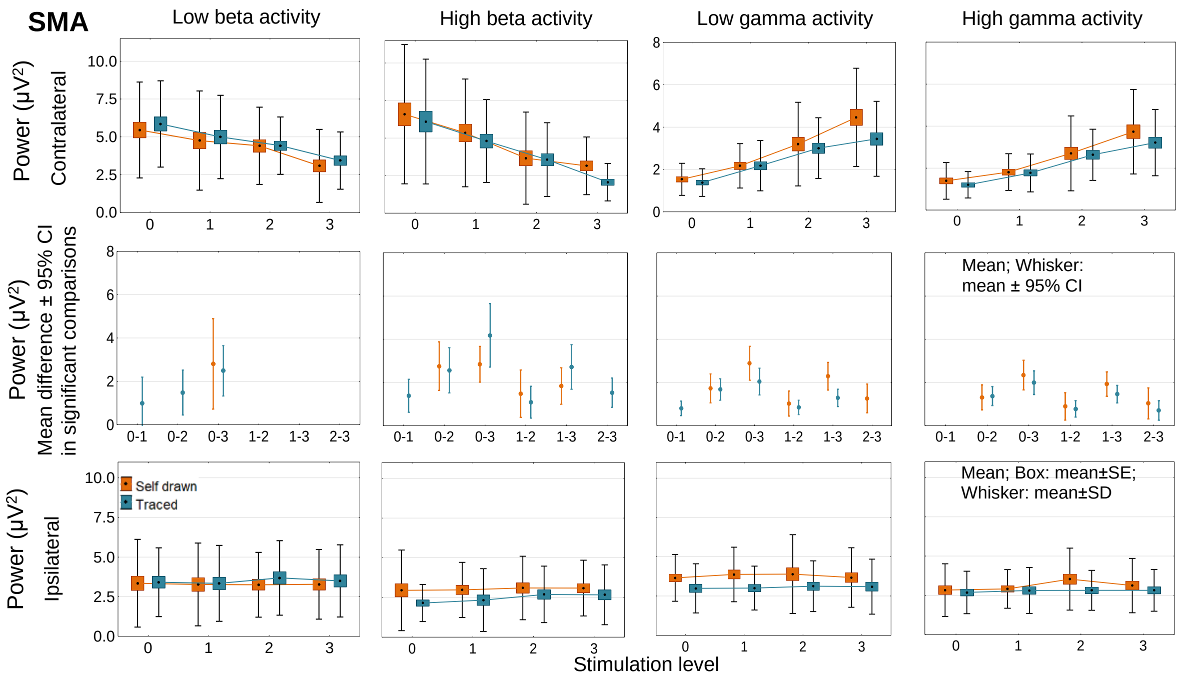

Supplementary Figure 1. Absolute beta and gamma power in the supplementary motor area

Power value changes in the tested hemisphere induced by stimulation in the low beta, high beta, low gamma, and high gamma bands in SMA are similar to the changes in the primary motor cortex. Power differences in significant post hoc comparisons of stimulation level effect are presented in the middle row. Bottom row: beta and gamma power remained unchanged with rising stimulation intensity in the not-tested hemisphere (ipsilateral to the movement). SMA: supplementary motor area

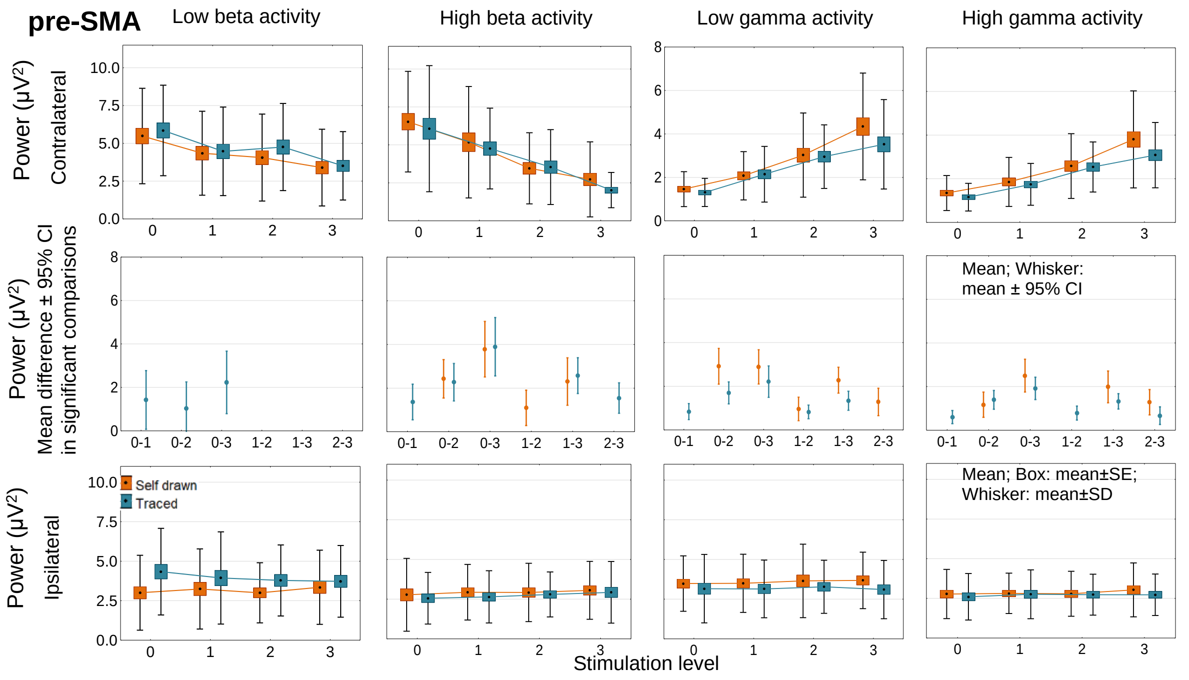

Supplementary Figure 2. Absolute beta and gamma power in the pre-supplementary motor area (pre-SMA)

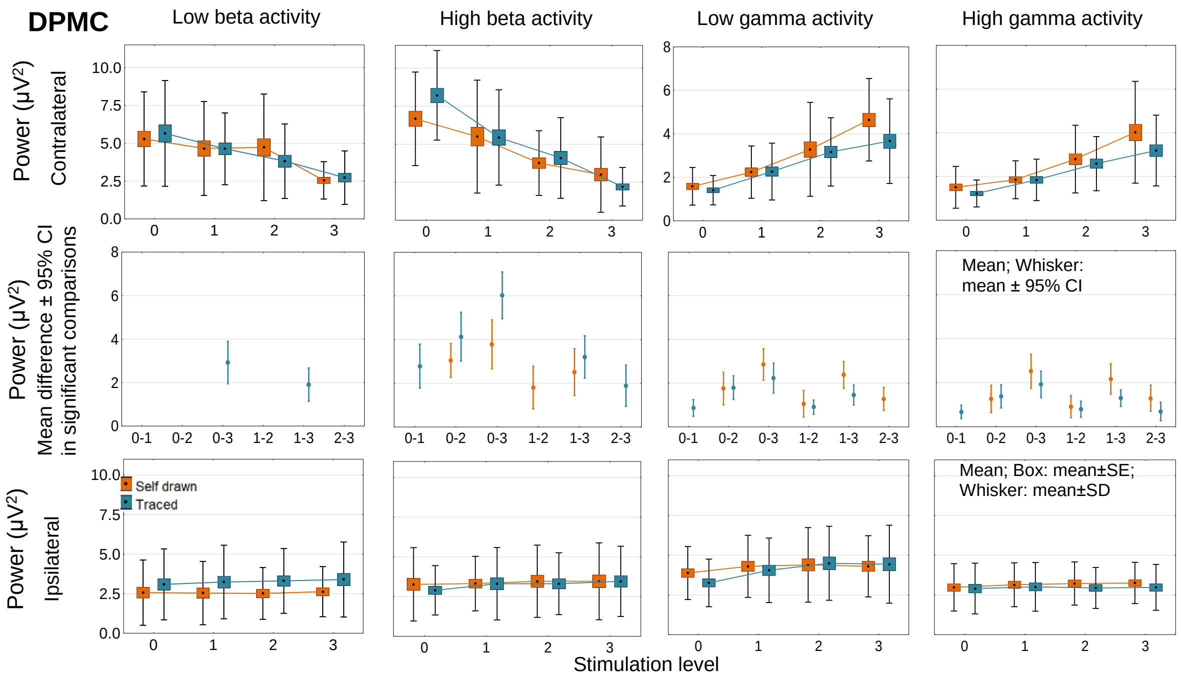

Supplementary Figure 3. Absolute beta and gamma power in the dorsal premotor cortex (DPMC)

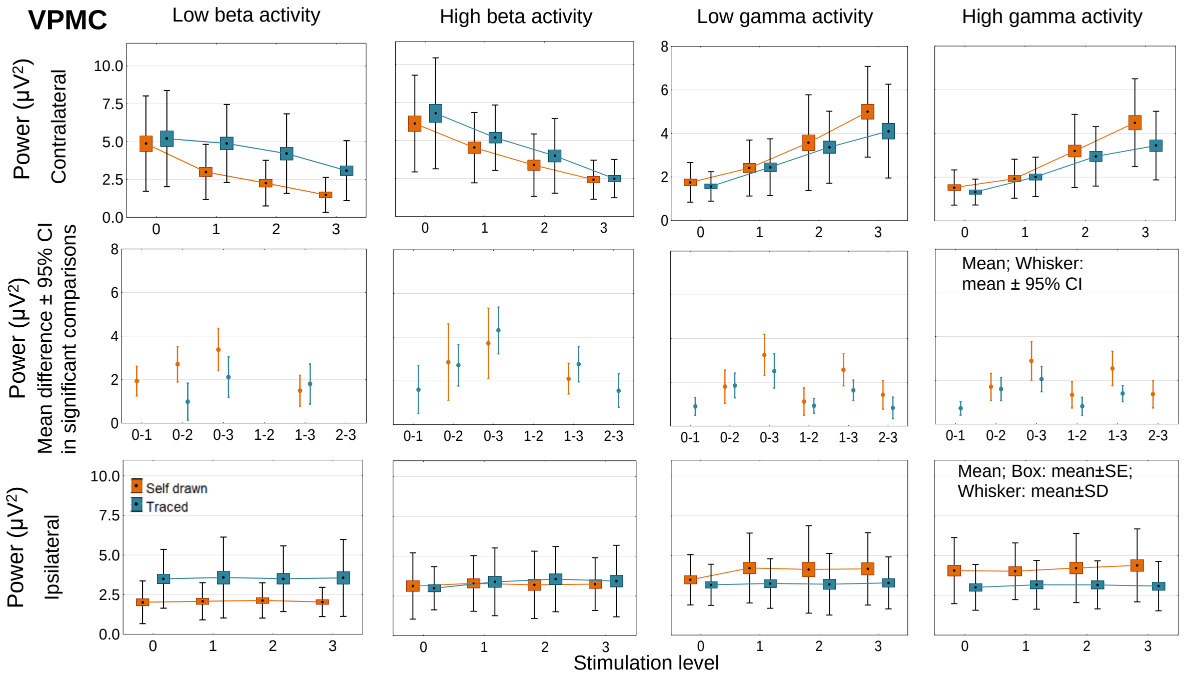

Supplementary Figure 4. Absolute beta and gamma power in the ventral premotor cortex (VPMC)

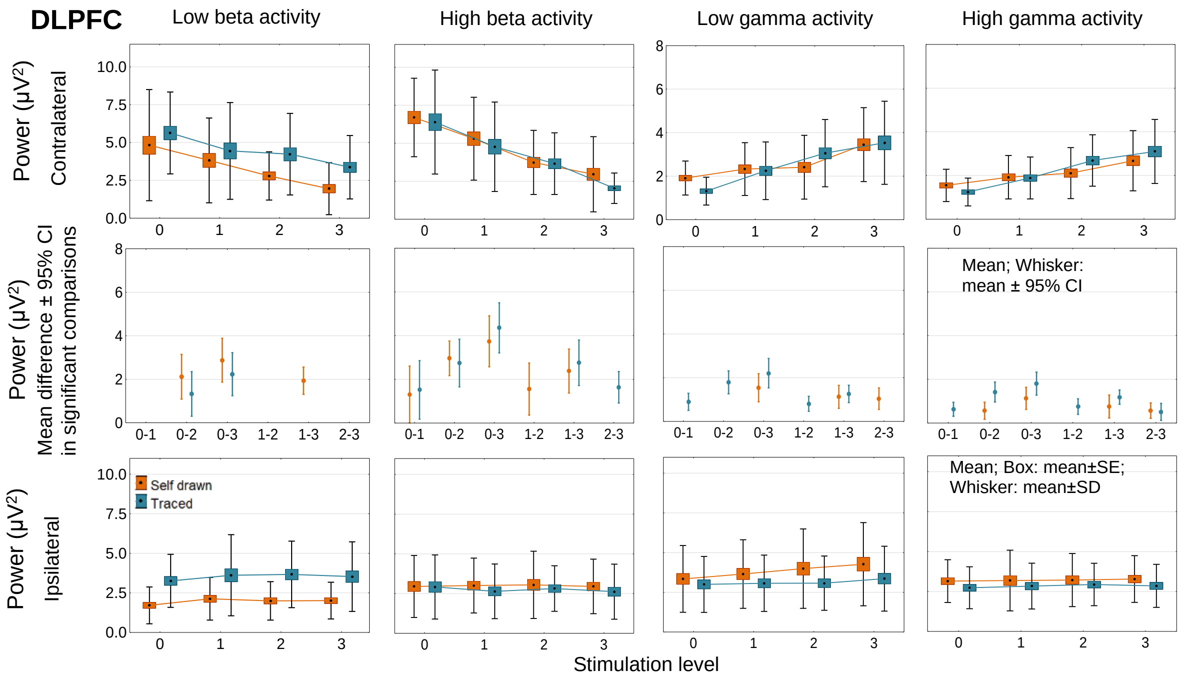

Supplementary Figure 5. Absolute beta and gamma power in the dorsolateral prefrontal cortex (DLPFC)

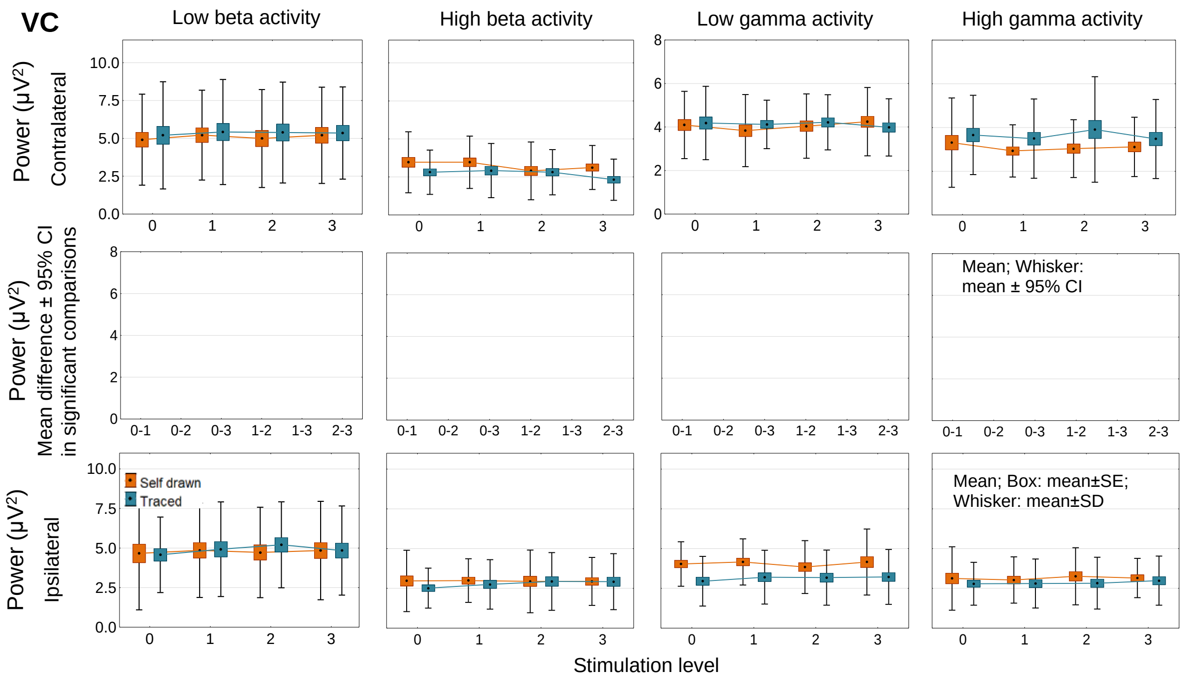

Supplementary Figure 6. Absolute beta and gamma power in the visual cortex
Subthalamic stimulation did not affect absolute power in any of the analyzed frequency bands in the visual cortex used as a reference region against the motor areas. VC: visual cortex

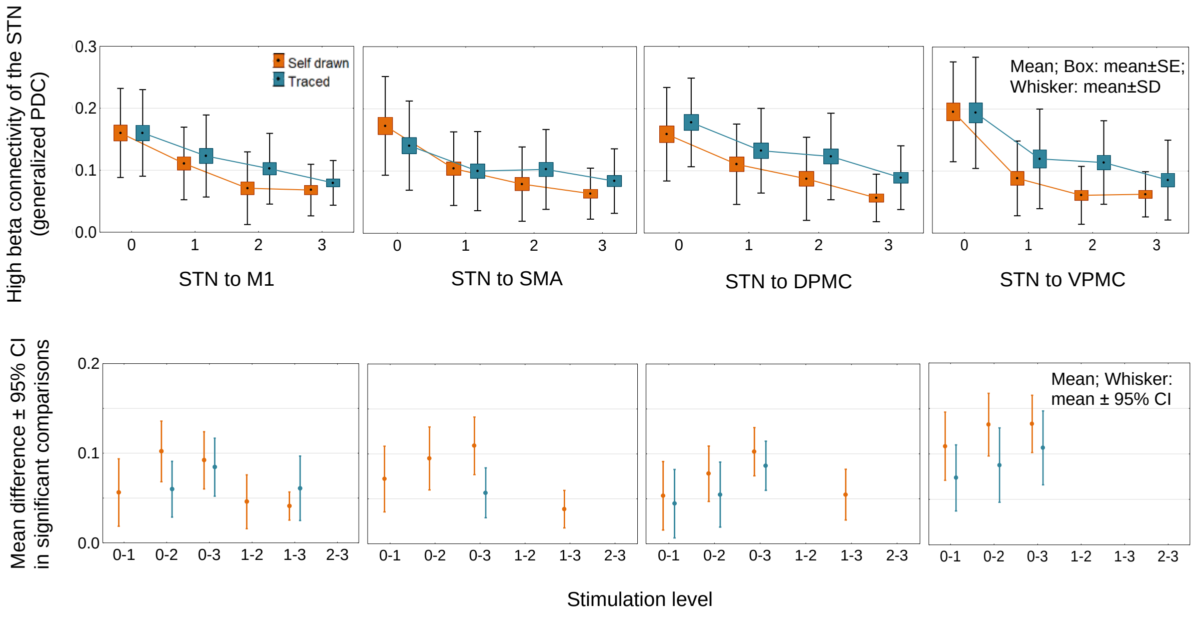

Supplementary Figure 7. Stimulation effects on hyperdirect high-beta connectivity: subthalamo-cortical direction

Effective connectivity between the STN and motor cortical areas decreased in the high beta frequency band with increasing stimulation intensity in a bidirectional manner. Cortico-subthalamic connectivity changes are shown in Figure 5; here, we present the decrease in effective subthalamo-cortical effective connectivity. Significant post hoc comparisons of stimulation level effect are presented in the bottom row. DPMC: dorsal premotor cortex, M1: primary motor cortex, SMA: supplementary motor cortex, STN: subthalamic nucleus, VPMC: ventral premotor cortex

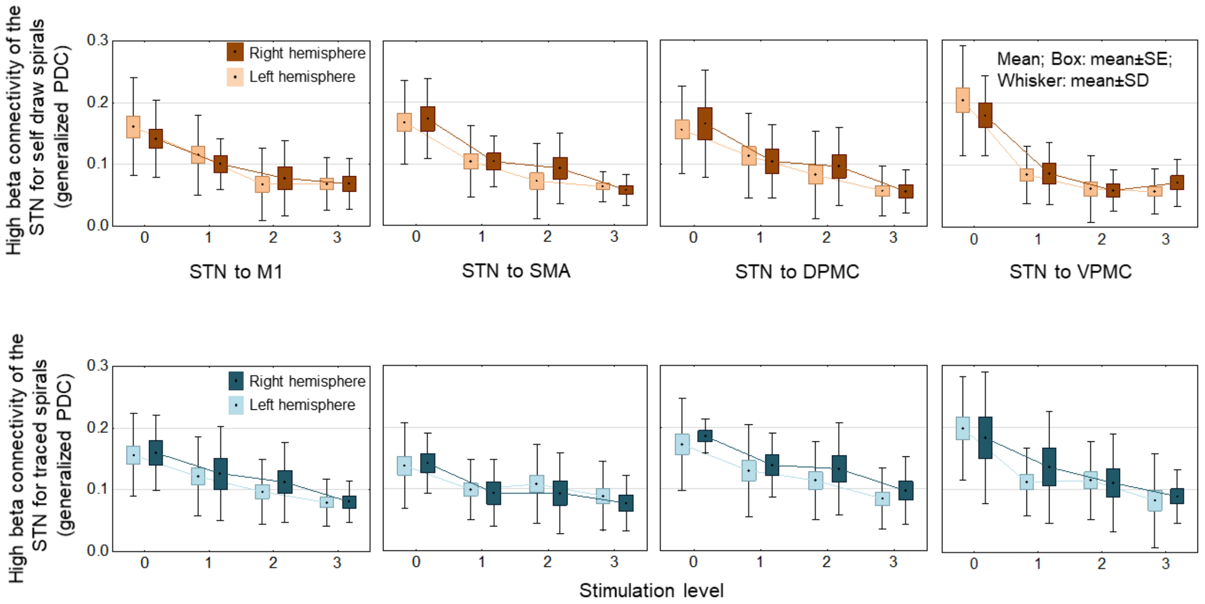

Supplementary Figure 8. Stimulation effects on hyperdirect high-beta connectivity according to the tested hemisphere: subthalamo-cortical direction

Effective connectivity between the STN and motor cortical areas decreased in the high beta frequency band with increasing stimulation intensity in a bidirectional manner regardless of the side of the tested hemisphere. Cortico-subthalamic connectivity changes are shown in Figure 6; here, we present the decrease in effective subthalamo-cortical effective connectivity. DPMC: dorsal premotor cortex, M1: primary motor cortex, SMA: supplementary motor cortex, STN: subthalamic nucleus, VPMC: ventral premotor cortex

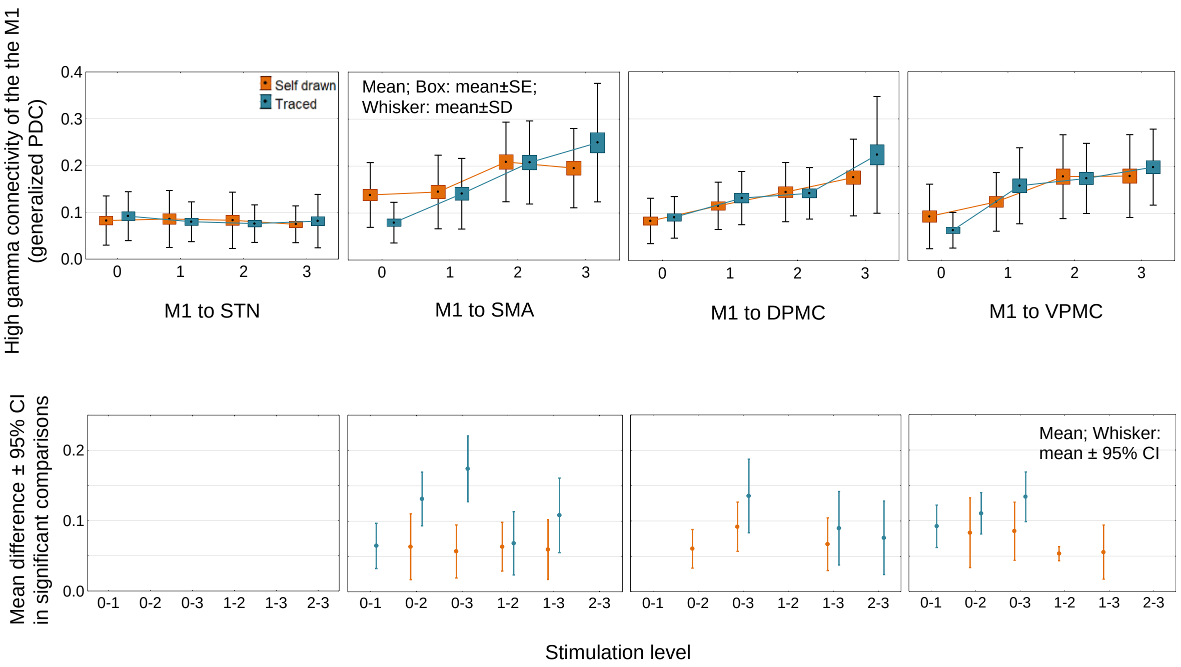

Supplementary Figure 9. Stimulation effects on task-related cortico-cortical and cortico-subthalamic high-gamma connectivity in the direction from M1 to the other structures

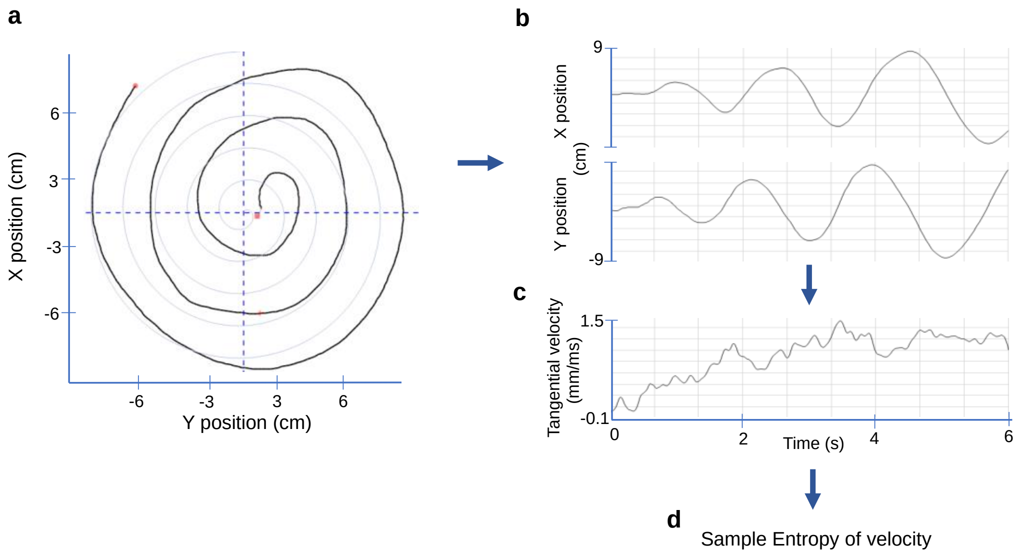

Supplementary Figure 10. Digitized spiral processing pipeline
a. Spiral acquisition and **b.** offline signal processing. For each spiral drawing, **c.** the average tangential velocity and **d.** the sample entropy of tangential velocity was calculated

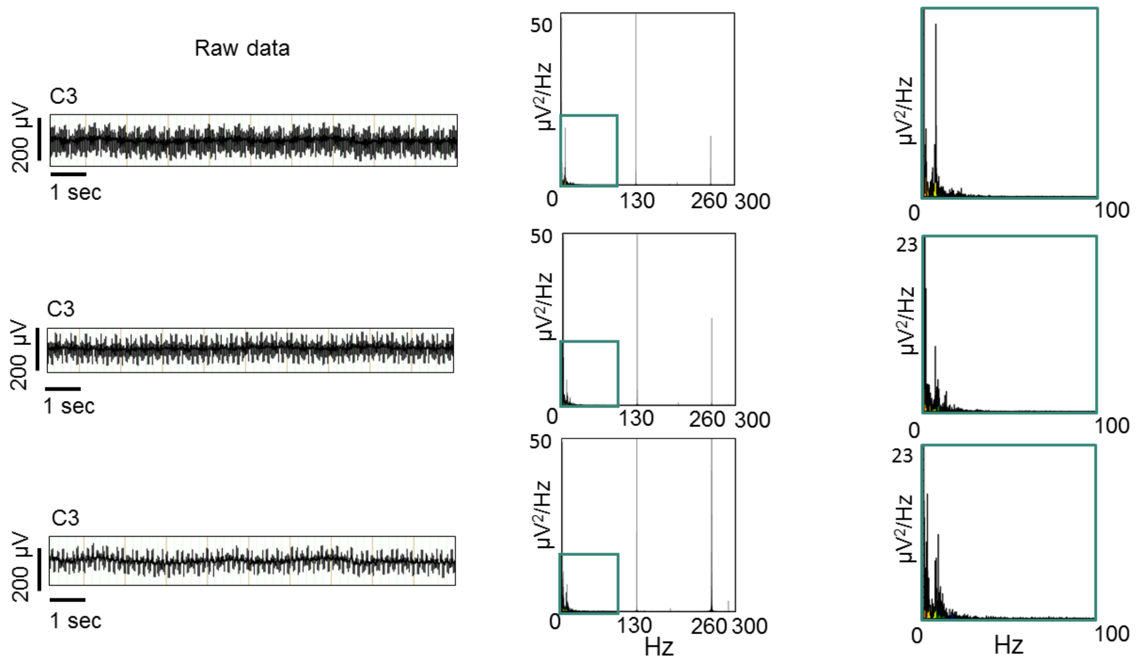

Supplementary Figure 11. EEG recording under maximum stimulation amplitude
Representative EEG time series and corresponding power spectral densities of non-saturated C3 channels from three patients were recorded during the maximum stimulation level of the left subthalamic nucleus. C3: central channel 3

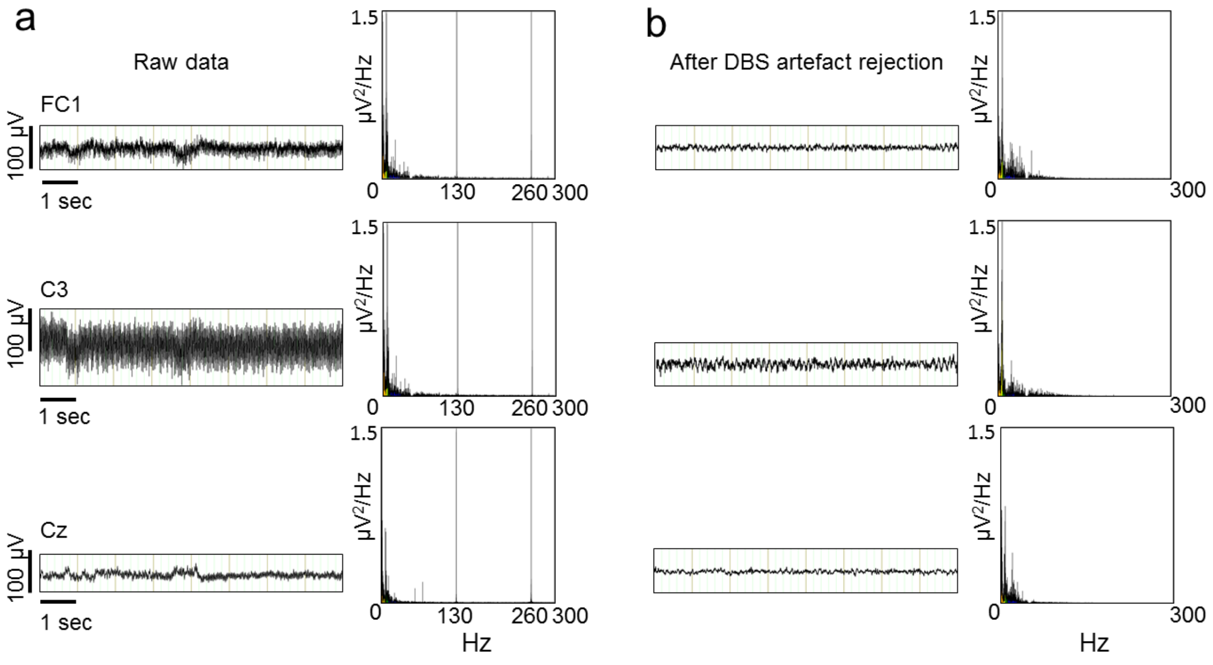

Supplementary Figure 12. EEG preprocessing pipeline
a. Representative notch-filtered time-series data from three EEG channels (FC1, C3, and Cz) of a patient receiving left STN-DBS during spiral drawing and its corresponding power spectral density (PSD) showing the power artifact created by the stimulation frequency (130 Hz) and its harmonic frequency (260 Hz). b. Cleaned time series after independent component analysis, DBS artifact rejection, and corresponding PSD. FC1: frontocentral channel 1, C3: central channel 3, Cz: midline central channel, DBS: deep brain stimulation, STN: subthalamic nucleus
